# Use of linseed oil to treat experimentally induced keratoconjunctivitis sicca in rabbits

**DOI:** 10.1186/1869-5760-3-4

**Published:** 2013-01-03

**Authors:** Magda Luzia Neves, Letícia Yamasaki, Osimar de Carvalho Sanches, Marcelo Sávio Paiva do Amaral, Helaine Stevanin, Rogério Giuffrida, Eudes Ramalho Candido, Jonatas Eduardo Góes, Luís Felipe da Costa Zulim, Augusto Schweigert, Rosimery Missuzu Fukui, Carlos Collares Meirelles, Carolina Akemi Sasaki, Silvia Franco Andrade

**Affiliations:** 1 Post-Graduate Program in Animal Science, University of Oeste Paulista (UNOESTE), Rodovia Raposo Tavares, Km 572, Presidente Prudente, São Paulo, 19001-970, Brazil; 2 Department of Anatomy Pathology, University of Oeste Paulista (UNOESTE), Rodovia Raposo Tavares, Km 572, Presidente Prudente, São Paulo, 19001-970, Brazil; 3 Department of Small Animal Surgery, University of Oeste Paulista (UNOESTE), Rodovia Raposo Tavares, Km 572, Presidente Prudente, São Paulo, 19001-970, Brazil; 4 Faculty of Veterinary, University of Oeste Paulista (UNOESTE), Rodovia Raposo Tavares, Km 572, Presidente Prudente, São Paulo, 19001-970, Brazil; 5 Veterinary Hospital, University of Oeste Paulista (UNOESTE), Rodovia Raposo Tavares, Km 572, Presidente Prudente, São Paulo, 19001-970, Brazil; 6 Department of Small Animal Medicine, University of Oeste Paulista (UNOESTE), Rodovia Raposo Tavares, Km 572, Presidente Prudente, São Paulo, 19001-970, Brazil

**Keywords:** Histopathology, Keratoconjunctivitis sicca, Linseed oil, Omega 3 and 6, Rabbits

## Abstract

**Background:**

The objective of this study was to evaluate the effectiveness of various linseed oil (LO) preparations (oral, topical, oral and topical combined) in treating experimentally induced keratoconjunctivitis sicca (KCS) in rabbits. Twenty male New Zealand white rabbits were divided into four groups: group C (control), group OLO (oral LO), group TLO (topical LO), and group OTLO (oral and topical LO). The animals were evaluated weekly using Schirmer's tear test (STT), fluorescein test (FT), and Rose Bengal test (RBT) and were euthanized at the end of the experiment for histopathological analysis.

**Findings:**

There were significant improvements in the parameters analyzed (STT, FT, and RBT) and in the histopathological finding in all of the groups using LO.

**Conclusions:**

The analyzed results demonstrate that LO, administered orally or topically, was effective in treating experimentally induced KCS in rabbits, although combined oral and topical LO did not show additional benefits greater than those with a single route of administration.

## Findings

### Introduction

Keratoconjunctivitis sicca (KCS), or dry eye syndrome, is characterized by chronic inflammation of the cornea, conjunctiva, and lacrimal glands caused by quantitative and/or qualitative tear changes [1]. Recently, several studies have proven that essential fatty acids (EFAs), omega-3 (ω-3) and omega-6 (ω-6), administered orally are an alternative therapy for patients with various types of tear deficiency, including Sjögren's syndrome and KCS [2-6]. Additionally, the topical use of ω-3 and ω-6 effectively controlled the signs of experimentally induced inflammation in rats with KCS [7].

Eicosanoids are biologically active substances that regulate physiological processes and are lipid mediators of inflammation. From the ω-6 EFA-derived prostanoids, arachidonic acid, via cyclooxygenase 1 (COX1) pro-inflammatory mediators, is synthesized, including prostaglandin (PGE2), thromboxane (TXA2), prostacyclin (PGI2), and the leukotrienes (LTA4, LTB4, LTC4, and LTD4). However, non-inflammatory mediators (PGE1 and TXA1) are also synthesized from the EFA series of ω-6 by the action of COX1 on dihomo-γ-linoleic acid (DGLA) and from the EFA series of ω-3 by the action of COX1 on eicosapentaenoic acid (PGE3, PGI3, and TXA3) as well as by the actions of lipoxygenase (LTA5, LTB5, LTC5, and LTD5) and of docosahexaenoic acid [2,4].

The ω-3 and ω-6 EFAs are found in many foods, such as nuts, cold-water fish, soybeans, rapeseed oil, and linseed oil. Linseed, *Linum usitatissimum*, is composed of 57% ω-3, 16% ω-6, 28% monounsaturated fatty acids, and only 9% unsaturated fatty acids, and the ω-3-to-ω-6 ratio of 1:3 is considered close to ideal. Linseed is considered to be a natural anti-inflammatory agent due its potential in synthesizing non-inflammatory mediators, such as PGE1 and TXA1 [8-10]. The objective of this study was to evaluate the effectiveness of various linseed oil (LO) preparations (oral, topical, oral and topical combined), which are rich in ω-3 and ω-6, in treating experimentally induced KCS in rabbits.

## Materials and methods

Twenty male New Zealand white rabbits (*Oryctolagus cuniculus*) were housed in individual metal cages with water and food *ad libitum*. All of the procedures were approved by the Ethics Committee on the Use of Animals of UNOESTE under protocol no. 014/09. The induction model of KCS in rabbits was based on a previously published study [11], which described the removal of the lacrimal glands and nictitating membranes and the administration of atropine sulfate 1% eye drops TID until the confirmation of KCS diagnosis (≤5 mm/min Schirmer's tear test (STT) and/or Rose Bengal test (RBT) positive) and then throughout the treatment period (12 weeks) for KCS maintenance. The rabbits were evaluated at M0 (prior to the KCS induction surgery), M1 (1 week after the induction of KCS and the beginning of treatment), and M2 to M12 (i.e., assessments as weekly intervals beginning 1 week after the initiation of treatment). Of the 20 rabbits, 15 were induced using the KCS protocol described above and 5 were allocated to the control group without KCS induction. A week after the induction of KCS, the groups of animals were treated for 12 weeks as follows: group C (*n* = 5, control, one drop of placebo, 0.9% NaCl solution, topically twice a day (BID) in both eyes), group OLO (*n* = 5, 1 g/day of liquid LO made by Laboratory Ophthalmos-SP (São Paulo, Brazil) orally), group TLO (*n* = 5, one drop of LO eye drops made by Laboratory Ophthalmos-SP BID in both eyes), and group OTLO (*n* = 5, 1 g/day of liquid LO orally with one drop of LO eye drops BID in both eyes). STT was performed without anesthetic eye drops and was considered positive if the KCS values were ≤5 mm/min. The fluorescein test (FT) was performed to observe ulcers colored with 1% fluorescein strips moistened with a small amount of normal saline; they were scored 1 (negative for a corneal ulcer) or 2 (positive for a corneal ulcer). RBT was performed on stained cells devitalized by KCS; after instilling the anesthetic eye drops, we used one drop of 0.5% Rose Bengal, and they were scored 1 (the absence of stained cells devitalized by KCS) or 2 (the presence of stained cells devitalized by KCS). For the histopathological analysis at the end of the experiment (M12), the rabbits were euthanized using 2.5% IV sodium thiopental (200 mg/kg); after transpalpebral enucleation, the eyeball was placed in a solution of 10% formaldehyde for 24 to 48 h. The eyes were stored in 70% alcohol and were processed according to the technique for inclusion in paraffin; 5-μm-thick cuts of the cornea and conjunctiva were obtained, which were colored with hematoxylin and eosin (HE) as well as periodic acid-Schiff (PAS). For STT variables and the density of caliciform cells, we used the variance analysis test for paired samples in contrast to Tukey's method. For FT, RBT, and histopathology, a non-parametric Friedman test was used to compare the various times, and the Kruskal-Wallis test with Dunn's method was used for between-group comparisons. We adopted a significance level of *P* < 0.05.

### Results

**Figure 1:**
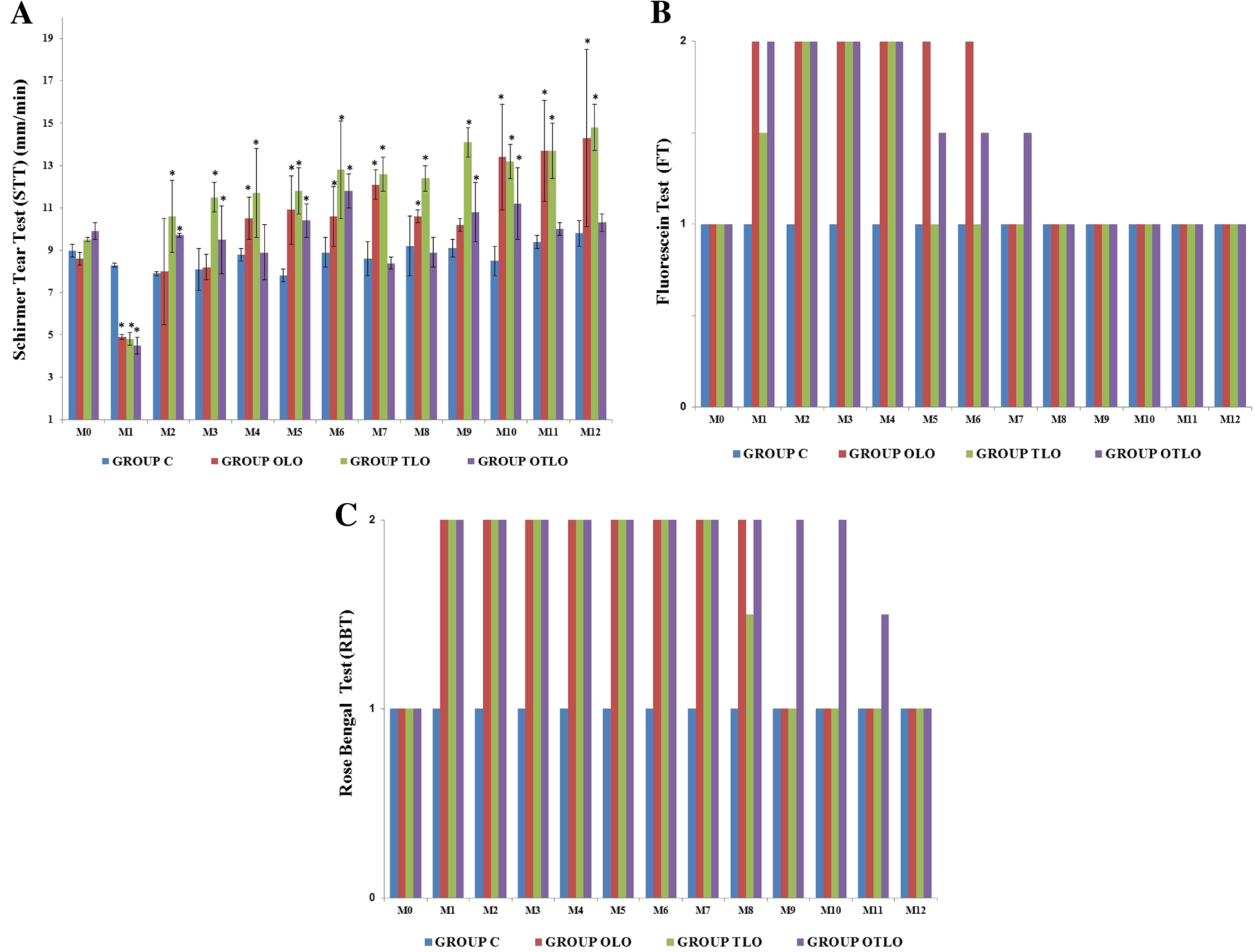
**STT, FT, and RBT results.** (**A**) Mean (±SD) STT values: ≤5 mm/min (KCS positive). (**B**) Median (quartiles P25 and P75) FT values: 1 (negative - absence of corneal ulcers) and 2 (positive - presence of corneal ulcers). (**C**) Median (quartiles P25 and P75) RBT values: 1 (absence of stained cells devitalized by KCS) and 2 (presence of stained cells devitalized by KCS). Group C (control), group OLO (oral LO), group OLT (topical LO), and group OTLO (oral + topical LO). The asterisk indicates *P* < 0.05 (Tukey's test compared to M0).

**Figure 2:**
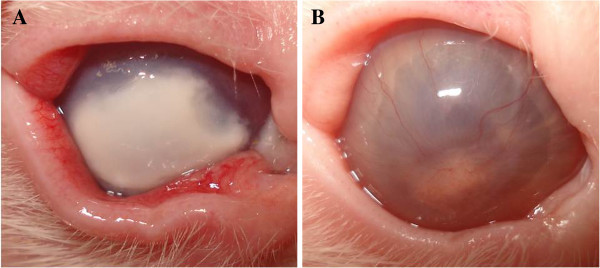
**Rabbit no. 4 from group OTLO (oral and topical LO).** (**A**) A melting ulcer in M1. (**B**) Partial resolution of the melting ulcer processes in M12.

**Figure 3:**
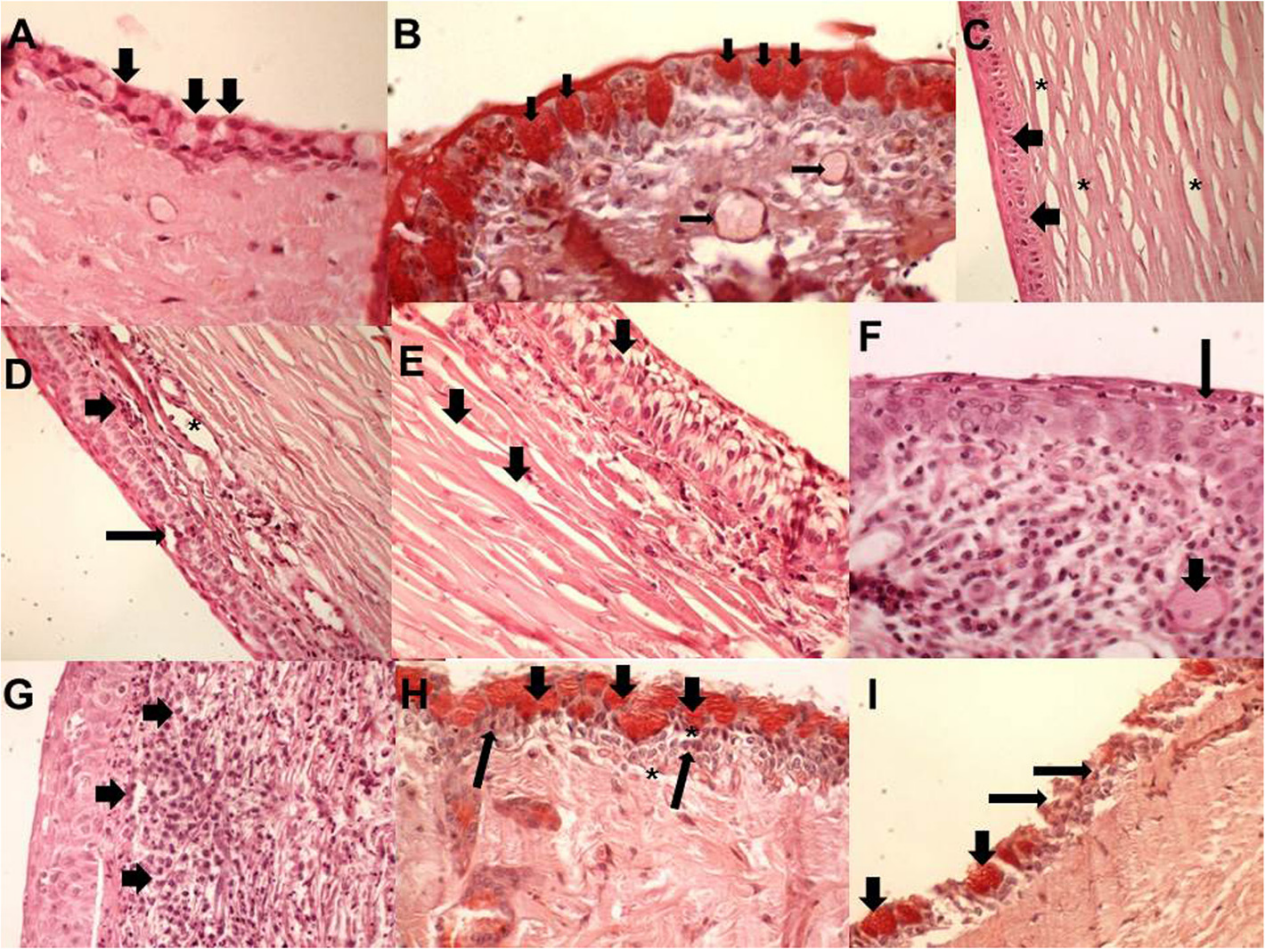
**Photomicrography of the cornea and conjunctiva.** (**A**) Group C without conjunctival inflammation; caliciform cells were seen in a mass (arrows); PAS, ×100. (**B**) Group OLO with caliciform cells (large arrows) and subconjunctival edema (thin arrows); PAS, ×100. (**C**) Group TLO with mild edema of the epithelium (arrows) and corneal stroma (asterisks); HE, ×100. (**D**) Group TLO with the corneal epithelium showing moderate edema (thin arrow) and the subcorneal area showing moderate inflammatory infiltration with lymphocytes (large arrow) and stroma in the blood vessel (asterisk); HE, ×100. (**E**) Group TLO with severe edema in the corneal and subcorneal epithelium and moderate edema in the stroma (large arrows); HE, ×400. (**F**) Group TLO with exocytosis of neutrophils in the corneal epithelium and with the presence of neutrophils (thin arrow) and congestion of blood vessels in the stroma (large arrow); HE, ×400. (**G**) Corneal epithelium showing exocytosis of neutrophils (arrows) and stroma showing intense edema; HE, ×400. (**H**) Group OTLO shows the presence of caliciform cells (large arrows), mild inflammatory infiltration (thin arrows), and subconjunctival edema (asterisks); PAS, ×400. (**I**) The conjunctiva of group OTLO group showing the presence of caliciform cells (large arrows) and lymphocytic inflammatory infiltration with conjunctival necrosis (thin arrows); PAS, ×100.

The STT, FT, and RBT results are shown in Figure 1. An interesting clinical finding was from rabbit no. 4 of group OTLO (oral and topical LO), which presented a melting ulcer at M1, and there was a partial resolution of the process until the end of the experiment (Figure 2). The histopathology results of the cornea and conjunctiva in group C showed no changes (Figure 3A). Upon histopathology of the cornea, there was slight edema in group OLO (Figure 3B) compared to group TLO, which presented moderate edema (Figure 3C,D,E). In group TLO, the presence of inflammatory infiltrates with exocytosis of neutrophils was observed (Figure 3F,G). In the histopathological results of the conjunctiva, the OLO (Figure 3B) and OTLO (Figure 3H) groups had mild and moderate edema, respectively, whereas group TLO had severe edema. The OLO and OTLO groups (Figure 3I) showed mild necrosis, whereas group TLO showed moderate necrosis. There was no statistical significance (*P* > 0.05) in the difference in the caliciform or goblet cell density in the conjunctiva among the groups, and the values were 9.8 ± 1.9 (C), 9.5 ± 2.3 (OLO), 10.7 ± 2.7 (TLO), and 9.0 ± 3.8 (OTLO).

### Discussion and Conclusions

The group treated with oral LO (group OLO) had normal tear production in the second week after starting treatment and a significant increase above the control group after 3 weeks. This finding is consistent with several studies, especially in patients with Sjögren's syndrome, in whom oral ω-3 and ω-6 supplementation has been recommended to control KCS symptoms [2-4,12]. In this study, topical LO was more effective in the early treatment of KCS, increased production of tears, and corneal ulcer resolution. The lipid mediators play an important role in the resolution and repair of corneal injuries [7,12,13].

The LO mechanism of action might be anti-inflammatory; the inhibitory response of the arachidonic acid inflammatory cascade has been proven to synthesize the non-inflammatory mediators PGE1 and TXA1 from ω-6 series by the action of COX1 on DGLA, which likely reduces the inflammation on the cornea, conjunctiva, and lacrimal glands; increases the production of tears; and improves the activity of caliciform cells, consequently causing an increase in the production of mucin. This increased mucin provides an improvement in the PTF quality, which leads to reduced tear evaporation in the aqueous portion of the eye [2,4,6,7,12]. In this study, LO topical instillation did not induce any ocular side effects, such as irritation, hyperemia, or discomfort. In addition, the concomitant use of LO orally and topically did not show benefits beyond a single route of administration. A hypothesis for this result is that an excess of ω-3 and ω-6 results in an improper ratio of these two EFAs. More pharmacokinetic and pharmacodynamic studies of oral LO and of LO in ocular topical formulations should be conducted to clarify these results. Therefore, we conclude that LO, orally and topically, was effective in treating experimentally induced KCS in rabbits, although combined oral and topical LO did not demonstrate additional benefits beyond those from a single route of administration.

## Competing interests

The authors declare that they have no competing interests.

## Authors’ contributions

MLN participated in the design of the study, acquisition, analysis and interpretation of data. LY and OCS participated in the histopathological analysisMSPA and HS participated in the surgery of the removal of lacrimal glands and nictitating membranes of the rabbit. sRG participated in the design of the study and performed the statistical analysis. ERC, JEG, LFCZ, AS, RMF, CCM and CAS participated in the ophthalmic exams and transpalpebral enucleation for material collection for histopathological analysis. SFA conceived of the study, and participated in its design and coordination and helped to draft the manuscript. All authors read and approved the final manuscript.
